# Navigating Industry 4.0: Leveraging additive technologies for competitive advantage in Colombian aerospace and manufacturing industries

**DOI:** 10.1371/journal.pone.0318339

**Published:** 2025-02-10

**Authors:** Thomas Tegethoff, Ricardo Santa, Juan Manuel Bucheli, Benjamin Cabrera, Annibal Scavarda

**Affiliations:** 1 Colegio de Estudios Superiores de Administración – CESA, Bogotá, Colombia; 2 Institución Universitaria Escuela Nacional del Deporte, Cali, Colombia; 3 Universidad Icesi, Cali, Colombia; 4 Federal University of the State of Rio de Janeiro, Rio de Janeiro, Brazil; Poznań University of Technology, POLAND

## Abstract

Industry 4.0 initiatives aim to improve competitive advantage. Additive technologies are a technological innovation and have great potential in a developing country like Colombia, The study analyzes how additive technologies, strategies, and process innovation interact to generate positive results, measured by the achievement of operational effectiveness in the aerospace sector and the manufacturing industry in general. Furthermore, the study explores the integration of additive technologies within the framework of Industry 4.0, focusing on the Colombian aerospace and manufacturing sectors. It investigates how these technologies, strategies, and process innovation contribute to operational effectiveness. Data were collected using a Likert-style questionnaire, developed based on established models of innovation framework and operational effectiveness model994 responses were obtained, of which 945 were deemed usable (423 from manufacturing and 522 from aerospace sectors). The analysis involved confirmatory factor analysis (CFA) and structural equation modeling (SEM) using SPSS and AMOS software, ensuring robustness through indicators like Cronbach’s Alpha and fit indices. The study compared findings across the two sectors to highlight differences in how additive technologies influence organizational outcomes. Initial results reveal that while additive technologies significantly enhance process innovation across sectors, their direct impact on operational effectiveness is evident only in the aerospace industry. The findings underscore that the aerospace sector benefits from additive technologies due to their need for complex, high-quality, and small-batch production, emphasizing their role in fostering precision and customization. However, the lack of impact in the manufacturing context is attributed to limited strategic alignment and inadequate implementation practices. Moreover, the results indicate that process innovation is a critical mediator, facilitating the translation of technological advancements into improved operational outcomes. Despite moderate correlations between strategies and operational effectiveness in manufacturing, aerospace organizations exhibit more substantial strategic alignment due to stringent performance demands. These findings highlight the importance of fostering a culture of innovation, adapting organizational strategies, and addressing structural challenges to maximize the benefits of additive technologies. The study contributes to understanding the differential adoption of Industry 4.0 technologies in developing economies, with implications for enhancing competitiveness and sustainability in global markets.

## 1. Introduction

Competitiveness is critical to an organization’s survival, profit generation, and contribution to the state’s economic, social, and political development [1,2]. Consequently, organizations strive to enhance their competitiveness through internal means or government assistance. A German government initiative – initially designed to increase the competitiveness of two of Germany’s most important industrial sectors and called Industry 4.0 - had a significant global impact [3].

The core concept of Industry 4.0 is digitizing the entire production chain, from suppliers to the end customer, minimizing human intervention throughout the value chain. Under these conditions, the goal is to combine production with high-quality services and intelligent monitoring and decision-making processes in a real-time environment. This self-organized production integrates people, machines, systems, products, and logistics through direct communication to make the entire organizational value chain more efficient [4,5].

Colombian organizations are aware that Industry 4.0 will affect them in some way, either positively by gaining more competitiveness through implementing Industry 4.0 elements or negatively by not doing so. The National Business Association of Colombia (ANDI) has been conducting surveys to estimate the state of Colombian organizations in implementing Industry 4.0 in the national territory. In their 2019 survey, 63.5% of their affiliates have a digital transformation strategy. 88.2% of organizations are familiar with the concept of Industry 4.0, and likewise, they are aware that the impact extends across the entire organization and not just a specific area (93.2%). However, this survey does not specify or evaluate the results of implementing Industry 4.0 initiatives in Colombian organizations. It is, therefore, essential to determine whether these initiatives have substantially impacted organizational results and whether their objectives have been achieved. Since Industry 4.0 encompasses a universe of possible initiatives to improve organizational results, this study focuses on determining the impact of additive technologies on strategies and changes in production processes to increase operational effectiveness. Deficient infrastructure, the Colombian topography, and the inherent potential for decentralized production in small batches through additive technologies generate advantages that Colombian organizations should exploit.

At the heart of Industry 4.0 lies the digitization of the entire production chain, from suppliers to end customers, with minimal human intervention. This transformation integrates intelligent monitoring, decision-making in real-time, and the seamless communication of people, machines, systems, and logistics throughout the value chain. The ultimate objective is to achieve a self-organized production system that optimizes efficiency, reduces waste, and improves product and service quality. As Industry 4.0 becomes a cornerstone of competitive strategy, it underscores the need for organizations to adapt and innovate to meet the demands of an increasingly interconnected and technology-driven world [6,7].

Integrating Industry 4.0 concepts, characterized by the seamless interplay of digital technologies, automation, and data exchange, is pivotal in enhancing competitiveness within the manufacturing landscape. In this transformative paradigm, additive technologies emerge as a critical enabler, fostering agility and innovation [8]. Industry 4.0-driven smart factories leverage additive manufacturing to facilitate on-demand production, rapid prototyping, and intricate customization, responding swiftly to dynamic market demands. The convergence of these technologies empowers organizations to achieve heightened operational efficiency, reduced lead times, and enhanced product quality [9]. Furthermore, the real-time data analytics inherent in Industry 4.0 enable predictive maintenance, optimizing the performance of additive manufacturing equipment. The synergy between Industry 4.0 and additive technologies elevates production capabilities and positions companies at the forefront of global competitiveness by fostering adaptive, cost-effective, and highly responsive manufacturing ecosystems [10].

Additive manufacturing (AM) has also revolutionized the aerospace industry by offering unprecedented design freedom, efficiency, and cost-effectiveness. Unlike traditional manufacturing methods, AM builds components layer by layer, enabling the creation of complex, lightweight structures that are critical for aerospace applications. This technology is particularly advantageous for producing intricate parts, such as turbine blades, fuel nozzles, and satellite components (such as antenna and communication devices), which require precision and reliability under extreme conditions. By minimizing material waste and allowing for the use of advanced materials like titanium alloys and high-performance polymers, AM significantly reduces production costs and environmental impact. Furthermore, the ability to rapidly prototype and customize designs facilitates innovation and accelerates development timelines, ensuring that aerospace manufacturers can meet evolving demands. AM also supports the concept of on-demand manufacturing, which is particularly beneficial for remote operations, such as space missions, where spare parts can be produced in situ. As the aerospace industry continues to prioritize efficiency, sustainability, and performance, additive manufacturing is set to remain a cornerstone of its innovation strategy. Consequently, the integration of additive manufacturing into antenna and communication system production not only drives innovation and cost-effectiveness in the aerospace sector but also addresses critical challenges in scalability, performance, and adaptability [11–14].

Many studies focus on the impact of strategies on the implementation of new technologies and the organization’s processes [15–17]. Nevertheless, the impact of process changes and innovation on organizational strategy still requires a more profound understanding [18,19].

The findings of this research are expected to contribute to both theoretical and practical understanding. Theoretically, the study aims to bridge gaps in the literature by exploring the dynamic interplay between additive technologies, strategy, and process innovation in the context of developing economies. Practically, it provides actionable recommendations for Colombian organizations seeking to enhance their competitiveness through Industry 4.0 initiatives. By identifying the conditions under which additive technologies contribute to operational effectiveness, the study offers a roadmap for leveraging these innovations to achieve sustainable growth.

Consequently, the research question for this study is: “What impact do factors such as innovation strategies and process innovation have on the relationship between additive technologies and operational effectiveness? Additionally, this question will be answered using two samples, one from the manufacturing industry and the other from the aerospace industry. This question will be answered using structural equation methodology, with input from collaborators from various Colombian organizations.

The remainder of the article is structured as follows. The conceptual framework for developing hypotheses and presenting the conceptual model of the relationship between the suggested variables is provided in Section 2, and the research methodology is presented in Section 3. The most significant observations and outcomes are presented in the fourth part. The used measurement items are shown in Section 5. Finally, in Section 6, the study’s conclusions, limitations, and potential directions for future research are reviewed.

## 2. Theoretical framework

### 2.1. Operational effectiveness and competitiveness

Competitiveness is an essential element for the survival of any organization and, therefore, must be a fundamental part of business strategies [20,21]. Consequently, it is essential to define the competitive advantage that the organization has over its competitors. The World Economic Forum (WEF) considers competitiveness to determine a country’s productivity. These factors include institutions, policies, infrastructure, and society. Likewise, the World Competitiveness Center proposes that competitiveness is how countries, regions, and organizations manage and organize their competencies and skills to achieve long-term economic growth and increase well-being.

Therefore, it is crucial to highlight the three fundamental aspects of competitiveness. The first objective is the nation’s well-being or the organization’s survival. Secondly is the nation’s or organization’s ability to produce and distribute goods and services—finally, the method of measuring competitiveness for the country and the organization [1,22]. Operational effectiveness is not the same as an organization’s strategy; both components are necessary to gain a competitive advantage, but they have different elements. The strategy involves carrying out activities and routines different from those of competitors or, if similar activities are executed, performing these activities differently. In contrast, operational effectiveness involves performing the same activities as the competition but more efficiently and superiorly [20,23]. Operational effectiveness has five dimensions that allow for measuring its impact on the organization [24,25]:

#### 2.1.1. Quality.

Quality is one of the most essential concepts in user satisfaction. It means giving customers what they need or want at the right time. Additionally, from an organizational perspective, quality also means producing without defects. Consequently, most organizations, regardless of their production processes, manage concepts such as delivery times, after-sales services, delivery times, warranties, or service forms, among others, to satisfy the customer and offer the best quality they expect [26,27].

#### 2.1.2. Speed.

Speed is the response time needed to meet customer requirements or to develop a new product or service. The organization also can respond to legal changes, market changes, and customer needs. Therefore, speed is essential in gaining a competitive advantage, as it allows a rapid response to any situation [26,28].

#### 2.1.3. Flexibility.

When discussing the concept of flexibility, it is essential to note that this term is closely linked to speed. Flexibility means responding on time to last-minute changes by the customer and the organization’s ability to anticipate market changes and make necessary adjustments. Similarly, flexibility includes changes in production processes, product or service designs, and any changes that may arise during the production process throughout the value chain [28,29].

#### 2.1.4. Reliability.

It is the fundamental element for the customer when acquiring the product. It refers to the product meeting its predetermined specifications under expected environmental parameters and fulfilling what the product or service provider promised. Reliability is one of the central elements in achieving customer satisfaction and quality [30,31].

#### 2.1.5. Cost.

Reducing costs is one of the most important goals of any organization. Cost reduction can be achieved by reducing waste, improving the efficiency of all organization processes and activities, and achieving organizational goals at the lowest possible cost. In this way, the organization can offer the product or service at a lower price to the customer or achieve a higher profit margin [32,33].

Achieving operational effectiveness requires strategically optimizing cost, quality, flexibility, reliability, and speed. By meticulously managing these key factors, organizations can enhance their overall performance, remain competitive in dynamic markets, and meet the ever-evolving demands of customers. Striking a balance among these pillars not only ensures efficient day-to-day operations but also positions businesses to adapt swiftly to changes, fostering resilience and sustained success in the long run [34].

### 2.2. Additive technologies

Additive technologies are a significant part of the Industry 4.0 concept due to their implications for decentralized production and the possibility of differentiated production in small batches and high complexity [4]. Additive technologies in production occur through 3D printing, layer by layer. The results are minimal to no waste and permit the generation of more complex shapes than subtractive technologies or traditional manufacturing [35]. The ongoing development of materials suitable for 3D printing, ranging from environmentally friendly materials to metal filaments, resins, nitinol (used in medicine), or carbon, enables a high degree of flexibility in 3D printing designs [36,37].

This transformative technology holds significant implications for competitiveness in the manufacturing sector. Additive manufacturing enhances operational efficiency and flexibility by enabling precise and customizable production with reduced material waste. Companies leveraging additive manufacturing can respond swiftly to changing market demands, produce complex designs, and optimize supply chains. The ability to iterate quickly on prototypes and efficiently produce small batches fosters innovation and cost-effectiveness, positioning organizations at a competitive advantage. In essence, additive manufacturing empowers businesses to streamline processes, reduce lead times, and stay agile in an ever-evolving marketplace, ultimately contributing to heightened competitiveness [38].

Therefore, additive technologies are considered a crucial element within the Industry 4.0 concept, especially when integrated into an automated production line with minimal human intervention [39]. Additive technologies have been evolving since the 1980s. Still, their field of application expanded significantly with the expiration of relevant patents, moving beyond the healthcare sector (e.g., organ printing) and into other manufacturing industry sectors, including the Aerospace sector. Today, applications span across various industries, from the production of simple items like toys to highly complex and high-quality components, such as aerospace parts. The aerospace industry, in particular, has benefited from this technology due to its need for highly complex and precise parts made from premium materials and in small batches [40,41].

Given the advantages of additive technologies, which aid in the creation of products with less waste and more efficiency, as well as much more complex designs and the ability to produce in small batches at a lesser cost, this research proposes based on the relationship between additive technologies and operational effectiveness that:

H1: Additive technologies have a positive and robust impact on operational effectiveness.

### 2.3. Strategies

Strategies are a fundamental element of any organization. A proper strategy defines the medium and long-term objectives the organization wants and how to achieve these objectives [6]. Organizations can have similar objectives, but the strategies to achieve these objectives may differ due to differences in capabilities, skills, and resources [42]. The concept of Industry 4.0 has profoundly impacted the design of organizational strategies. Changes in production, even if the goals and targets remain the same, require a change in strategies [18,19,43]. The organization’s capabilities and competencies change when implementing Industry 4.0 initiatives and achieving goals and objectives [42,44].

Creating a competitive advantage in the Industry 4.0 era requires an appropriate strategy that considers existing capabilities and resources or how to acquire the necessary skills if they do not exist within the organization. Thus, the strategy should consider using existing capabilities and developing programs and initiatives to acquire the necessary capabilities to generate benefits from Industry 4.0 initiatives [45].

As part of Industry 4.0, additive technologies require suitable strategies to achieve the expected outcomes from this new technology and generate changes in internal organizational processes, which must be integrated into the organizational strategy. Several studies emphasize the importance of an adequate strategy in implementing new technologies [15,17]. Nevertheless, the impact of such changes or innovation in shaping the organizational strategy is less explored [46]. Likewise, when implementing additive technologies within an organization, it changes its capabilities and skills, which creates a new strategic horizon for the organization [47,48]. As additive manufacturing revolutionizes traditional production methods, a well-defined organizational strategy becomes imperative for businesses looking to capitalize on these advancements. Strategic integration of additive technologies enables companies to optimize supply chains, reduce time-to-market, and enhance overall operational efficiency. By aligning organizational goals with the capabilities of additive manufacturing, businesses can unlock new avenues for product innovation, customization, and cost-effective production [49,50].

Additive technologies also have a positive and significant impact on organizational strategies because their adoption often necessitates a rethinking of existing business models and long-term objectives. Unlike traditional technologies that might align with pre-existing strategic frameworks, additive manufacturing introduces novel capabilities such as on-demand production, rapid prototyping, and customization, which inherently reshape an organization’s competitive positioning and operational priorities. These transformative capabilities push organizations to adapt their strategies to maximize the value derived from the technology, such as optimizing supply chains, leveraging decentralized production, and targeting niche markets with customized offerings. On the other hand, while strategies influence technology adoption, they often provide a guiding framework rather than directly driving the revolutionary changes enabled by additive technologies. Therefore, additive technologies act as a catalyst for strategic evolution, compelling organizations to redefine their goals and methods to align with the disruptive potential of these innovations [51,52]. Therefore, we propose that there is a relationship between additive technologies and organizational strategy:

H2: Additive technologies have a positive and significant impact on organizational strategies.

Similarly, strategies aim to improve the organization’s competitive advantage through operational effectiveness. Therefore, strategies must be designed to change processes, structures, and the acquisition and utilization of organizational resources, thus improving operational effectiveness [53,54]. A well-crafted strategy aligns the various components of an organization towards common goals, optimizing processes for efficiency and effectiveness. It is a guiding framework for decision-making, resource allocation, and performance measurement. A strategic approach ensures that operational initiatives are not isolated but contribute cohesively to overarching objectives. Whether it involves streamlining processes, adopting new technologies, or enhancing workforce capabilities, a clear strategy helps organizations prioritize and allocate resources judiciously. Moreover, in a rapidly changing business environment, a robust organizational strategy provides the agility to adapt to evolving market conditions and stay ahead in a competitive landscape, ensuring sustained operational excellence [55].

Organizational strategies play a pivotal role in leveraging additive technologies to enhance operational effectiveness by aligning technological capabilities with business objectives. A well-crafted strategy ensures the seamless integration of additive manufacturing into production processes, enabling organizations to optimize resources, reduce waste, and improve efficiency. By strategically adopting additive technologies, companies can streamline supply chains, accelerate product development cycles, and customize production to meet dynamic market demands. Furthermore, strategies that prioritize innovation foster the development of novel processes and products, enhancing competitiveness. When additive technologies are strategically implemented, they transform operations by enhancing reliability, flexibility, quality, and cost-effectiveness, ultimately driving superior organizational performance [56]. Consequently, there is a relationship between organizational strategies and operational effectiveness:

H3: Organizational strategies have a positive and significant impact on operational effectiveness.

### 2.4. Process innovation

Innovation is fundamental to any organizational strategy [57,58]. Industry 4.0 is a transformative innovation in managing production or offering services and is considered the fourth industrial revolution. It is necessary to generate, promote, and manage innovation adequately within the organization [59,60]. Therefore, innovation is considered the main driver of an organization’s competitiveness. Innovation management and innovative culture within an organization enable the improvement of process efficiency, market conquest, and the generation of innovative products and services, and, in general, support the organization’s survival over time and in markets [61,62]. Beyond managing an innovation process within the organization as something additional or not to be taken seriously, Industry 4.0 obliges organizations to rethink their business model, their way of managing their product and service offerings, and how to reach the customer, that is, to make innovative changes within the organization that improve its efficiency [5,63].

Depending on the context, the concept of innovation has different definitions. For Evangelista & Vezzani [64], innovation is any process that generates knowledge for the organization and its application within the organization. The Organization for Economic Cooperation and Development (OECD) extends the concept to include technological innovations and organizational innovations that involve changes from the organization’s structure to changes in procedures, routines, processes, and activities. Another definition considers innovation as any process or adaptation to improve the organization’s competitiveness [65,66]. The approach to innovation can be defined from the perspective of a result, a process, and a mindset of change. Considering these approaches, innovation, as a result, encompasses all changes in products, supply chains, the design of novel products or services, processes, and activities, or, in general, all results of the organization’s business model.

Similarly, innovation can also be considered a process, as it defines and designs organizational activities, processes, and routines to achieve the results of innovation initiatives. Finally, an innovative mindset within the organization is necessary, an innovative culture that allows for changes, experimentation, and acceptance that, in some cases, failure may occur [67,68]. Therefore, innovation is pervasive throughout the organization and cannot only be managed by a few individuals or departments. Constant support is required from management and all employees at all organizational levels.

Tidd & Bessant’s [28] model has been widely discussed as part of an organizational innovation model [69]. Tidd & Bessant [28] suggest an innovation space within which organizations can be located according to their way of innovating (radical or incremental) or in which organizational aspect they are innovating (product, position, processes, or paradigmatic). Industry 4.0 initiatives are primarily located in the process innovation space, as they modify the ways of producing and offering products and services to customers, even if the product or service remains the same. The product may be the same, but how it is produced and delivered to the customer changes to a more efficient, faster, and effective process [63,70]. Since additive technologies are an innovation that affects organizational processes to improve efficiency, the fourth hypothesis proposes a direct relationship between additive technologies and process innovation:

H4: Additive technologies have a positive and significant impact on process innovation.

If an organization changes its processes due to the inclusion of new technologies, these changes also affect its strategy and vice-versa [46]. Process innovation means a change in capabilities and competencies, and consequently, it opens up a new strategic horizon for the organization [71]. In this sense, the organization can redefine or refine its strategy depending on the process changes. These changes could improve the organization’s efficiency in production processes or the generation of services. A second possibility is that process innovation facilitates individualizing products or services to differentiate them from the competition and gain a competitive advantage [72,73]. Many studies analyze the impact of strategy on changes in organizational processes but also process changes can define and enhance organizational strategies [71,74]. Process innovation introduces new ways of working, which often expand an organization’s operational capabilities. Additionally, organizations that innovate processes are better equipped to adapt to changing market conditions. For instance, supply chain process innovations, such as just-in-time inventory management or blockchain for transparency, enable firms to respond swiftly to disruptions. These process improvements can redefine organizational strategies by emphasizing agility, risk management, and resilience in a dynamic global environment. Finally, process innovation can transform how organizations deliver value to their customers. For instance, innovations in digital payment systems or customer service automation redefine strategies by focusing on customer experience and convenience. These changes not only enhance operational efficiency but also align the organization’s strategic goals with evolving customer expectations [72,75–77].

Therefore, the following hypothesis proposes that:

H5: Process innovation has a positive and significant impact on organizational strategies.

One of the main objectives of making organizational changes, such as process innovation, is to improve the company’s results through more efficient processes and routines or by producing new products or services. These changes can be planned as part of a change in organizational strategies or improving production resources. Alternatively, they can respond to changes in the organization’s environment, such as changes in legislation, consumer preferences, or new technologies, among others [34,78]. Therefore, we propose the following hypothesis:

H6: Process innovation has a positive and significant impact on operational effectiveness.

**Fig 1:**
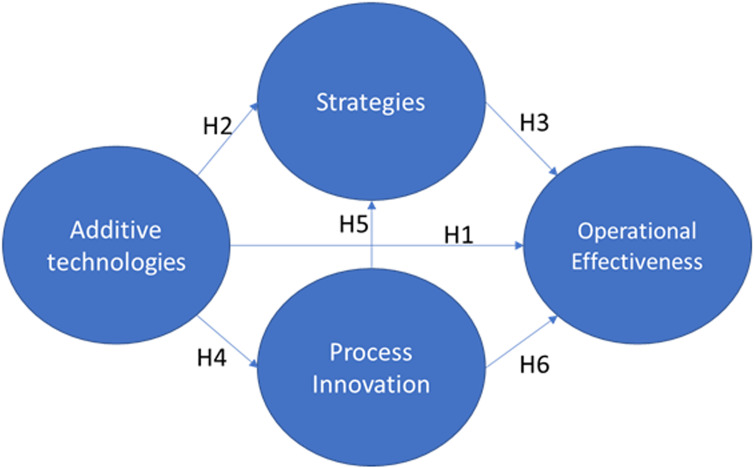
Hypothetical model.

Based on the literature review, we propose the following hypothesized model (Fig 1).

## 3. Methodology

This study adopts an exploratory approach. There is a scarcity of research on additive technologies as part of Industry 4.0 initiatives and their relationship with operational effectiveness, strategies, and process innovation in developing economies [39,56]. Exploratory studies are conducted when there is little to no existing evidence regarding the phenomenon under investigation, and the existing literature only provides an imprecise general idea related to the problem [79,80]. Exploratory research allows therefore for a deeper investigation into complex phenomena where existing theoretical frameworks provide limited guidance. By focusing on the aerospace and manufacturing sectors, this methodology captures a diverse range of industrial contexts, each with unique challenges and opportunities, thereby enriching the study’s findings and offering broader implications for Industry 4.0 adoption.

This study’s initial phase involves defining the critical variables to be considered and addressing issues in implementing Industry 4.0 that affect many Colombian organizations through an extensive literature review. A questionnaire assessed perceptions based on the variables of interest identified in the first phase. The survey instrument was meticulously designed, drawing on established frameworks, including Tidd & Bessant’s innovation model [28], Santa et al.’s operational effectiveness framework [81], and Porter’s strategic models[82] and concepts related to additive technologies and Industry 4.0 from Haleem & Javaid [39], Horst et al. [35], and Steenhuis & Pretorius [83] (13 questions).. Each model was selected to align with the study’s objectives, ensuring the robustness of the constructs measured. The Likert-style questionnaire encompassed 37 items across key dimensions such as additive technologies, strategies, process innovation, and operational effectiveness, enabling a comprehensive analysis of the interrelationships among these variables. Care was taken to validate the content of the survey through expert reviews and pilot testing, ensuring clarity and relevance to the industrial contexts under investigation. The Likert-style questionnaire (from strongly disagree to strongly agree) presented different statements based on the variables. Additionally, the questionnaire includes a demographic section to characterize the organization and the sector in which the surveyed individuals operate. This questionnaire was administered virtually due to the ongoing pandemic in the country, which made conducting in-person surveys complex. Additionally, leveraging the benefits of online surveys was considered [84,85]. Data collection started on July 2022 and finished in December 2023. At the beginning of the questionnaire, a paragraph is included to inform participants that by continuing to answer, they consent to the collection of their data. Participants are made aware that they can withdraw from the questionnaire at any point, despite their initial agreement to data collection by proceeding with the survey. The questionnaire was distributed to various organizational stakeholders, including managers, technologists, administrative personnel, and operational staff, as these individuals may have differing perceptions of the phenomenon under study [86,87]. The survey was approved by the Ethics committee of Colegio de Estudios Superiores de Administración – CESA.

**Fig 2:**
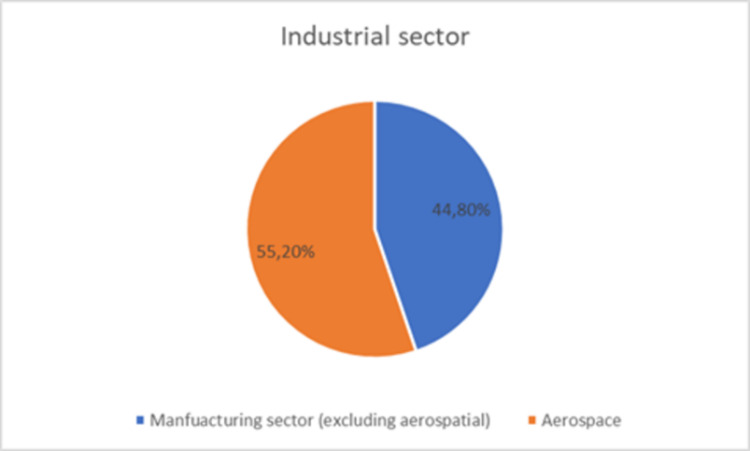
Sector of the respondent.

Of the 994 collected surveys, 945 (95%) were deemed usable. Most of these surveys were from the manufacturing sector (N =  423), followed by surveys collected from the aerospace industry (N =  522). Due to the significant interest of the aerospace sector in this study, the results of the two sectors are compared. By including a mix of stakeholders—ranging from managers to operational staff—the study captures a holistic perspective on additive technologies’ impact. The decision to focus on both the aerospace and manufacturing industries reflects their strategic importance in Colombia’s economy and their differing adoption patterns of Industry 4.0 initiatives. The aerospace sector’s emphasis on precision and high-quality standards contrasts with the broader manufacturing sector’s varied operational objectives, making this comparative analysis particularly valuable. Fig 2 shows the percentage of respondents working in the aerospacee or manufacturing sector.

**Fig 3:**
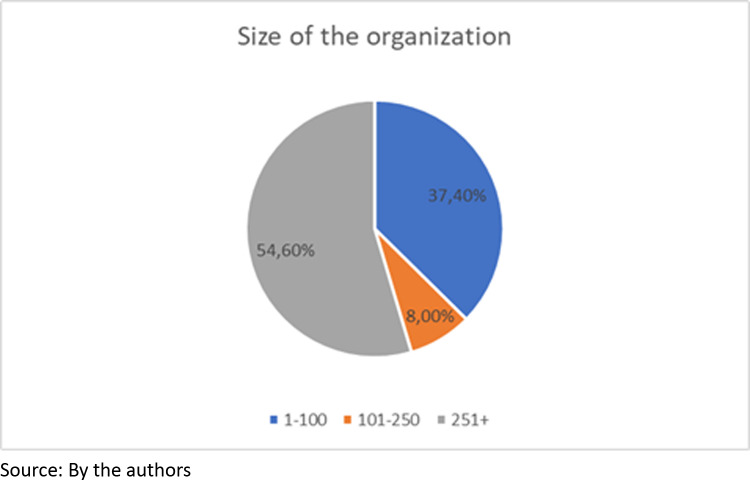
Organization size of the respondent.

Likewise, more than half of the respondents (54.6%) work in organizations with more than 251 employees, while only 8% work in organizations with 101 to 250 employees; meanwhile, 37.4% work in organizations with 100 or fewer employees (Fig 3).

The software packages SPSS and AMOS were employed to conduct the statistical or multivariate analysis. These programs were used for confirmatory factor analysis and structural equation modeling to measure the model’s robustness and the impact of the variables in the established model. Confirmatory factor analysis (CFA) was employed to validate the robustness of the model following the guidelines of Hair et al. [80] and Byrne [88]. The Cronbach’s Alpha indicator was evaluated following established parameters, and since all values exceed 0.7, it can be affirmed that internal consistency within the model is achieved [89,90]. The choice of these tools underscores the study’s methodological rigor, allowing for robust testing of hypotheses and the reliability of constructs. Lastly, the comparative analysis between the aerospace and manufacturing sectors adds depth to the methodology. By identifying sector-specific differences in the impact of additive technologies, strategies, and process innovation, the study offers nuanced insights into Industry 4.0 adoption. For instance, the aerospace sector’s reliance on high precision and stringent quality controls influences its strategic priorities differently than the broader manufacturing sector. This comparative perspective underscores the study’s contribution to understanding how industrial context shapes the outcomes of technological innovation.

Initially, the dataset was divided into two distinct groups: one comprising data from the aerospace industry and the other from non-aerospace industries. Subsequently, statistical analyses were conducted for each group separately to calculate Cronbach’s Alpha, ensuring the reliability of the constructs, and to evaluate model fit indices, thereby validating the robustness of the measurement models for both sectors. The model robustness indicators provide the following information:

In addition to assessing internal consistency through Cronbach’s alpha, this study employed Confirmatory Factor Analysis (CFA) to ensure the validity and reliability of the constructs. CFA was used to validate the measurement model by testing the relationships between observed variables and their underlying latent constructs. Fit indices such as the Comparative Fit Index (CFI), Tucker-Lewis Index (TLI), Normed Fit Index (NFI), and Root Mean Square Error of Approximation (RMSEA) were evaluated to confirm the robustness of the model. The results demonstrated strong model fit for both the aerospace and general manufacturing sectors, with CFI and TLI values exceeding 0.9 and RMSEA values below 0.08. These results indicate that the constructs were appropriately measured and aligned with the theoretical framework [91,92].

Considering the presented indicators (Tables 1 and 2), both models are robust and valid for conducting path diagram analysis within structural equation modeling to determine the impact and interrelation of the variables of interest.

**Table 1:** Cronbach’s alpha.

Variable	Ítems	Cronbach’s alpha	
		Aerospace sector	Manufacturing sector (except Aerospace)
Add. technologies	8	0.92	0.94
Strategies	6	0.91	0.93
Process innovation	4	0.91	0.94
Operational effectiveness	7	0.93	0.95

**Table 2:** Robustness indicators.

Indicator	Aerospace sector	Manufacturing sector (except Aerospace)
CMin/D.F. – Chi-square to degrees of freedom ratio	2.98	3.413
CFI – Comparative Fit Index	0.942	0.953
RMSEA – Root mean square error of approximation	0.06	0.059
NFI – Normed Fit Index	0.92	0.94
RFI – Relative Fit Index	0.90	0.93
IFI – Incremental Fit Index	0.94	0.95
TLI – Tucker-Lewis Index	0.93	0.93

## 4. Results

### 4.1. Results for the industry in general

**Fig 4:**
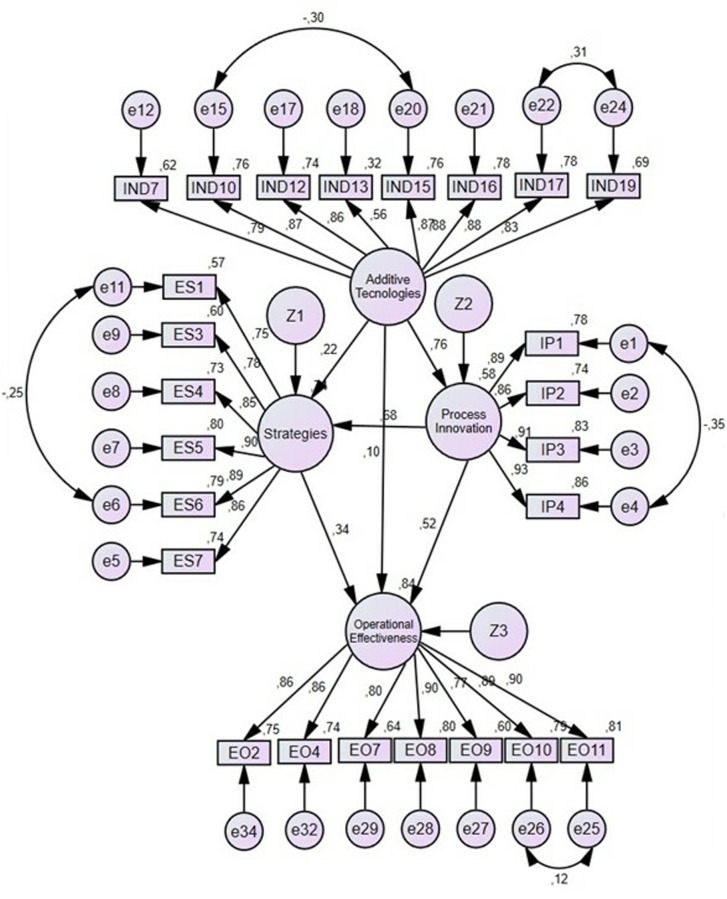
Structural model for the manufacturing sector (except Aerospace).

According to these results (Table 3 and Fig 4), only hypothesis 1 proposed in the theoretical framework is rejected. In this regard, additive technologies do not impact operational effectiveness (Hypothesis 1, β =  0.1; P =  0.05). According to the statistical analysis, there is a strong relationship between additive technologies and process innovation (Hypothesis 4, β =  0.76; P <  0.001), between process innovation and operational effectiveness (β =  0.52; P <  0.001), and between process innovation and organizational strategies (Hypothesis 5, β =  0.68; P <  0.001). Meanwhile, there is a moderate relationship between strategy and operational effectiveness (Hypothesis 3, β =  0.34; P <  0.001) and a weak relationship between additive technologies and strategies (Hypothesis 2, β =  0.22; P <  0.001).

**Table 3:** SEM results.

			Estimate	S.E.	C.R.	P	H
Operational Effectiveness	<-	Additive technologies	0.113	0.040	2,820	0.005	H1
Strategies	<-	Additive technologies	0.247	0.050	4,932	***	H2
Operational Effectiveness	<-	Strategies	0.326	0.047	6,982	***	H3
Process Innovation	<-	Additive technologies	0.888	0.051	17,312	***	H4
Strategies	<-	Process Innovation	0.665	0.048	13,925	***	H5
Operational Effectiveness	<-	Process Innovation	0.486	0.049	9,930	***	H6

### 4.2. Results for the aerospace sector

**Fig 5:**
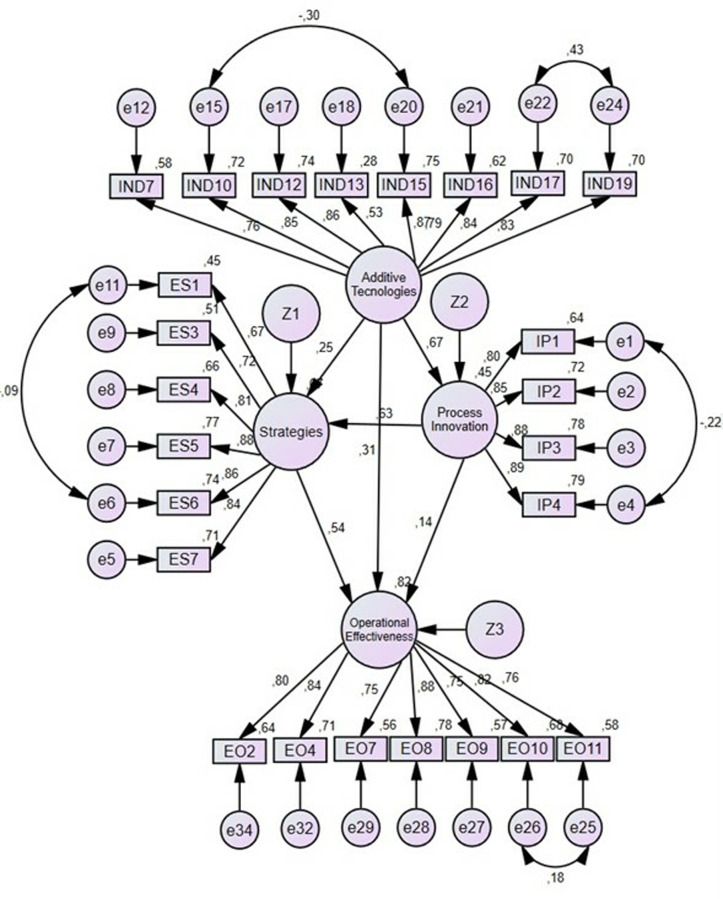
Structural model for the Aerospace sector.

According to the results (Table 4 and Fig 5), only hypothesis 6 proposed is rejected. Process innovation has no significant relationship with operational effectiveness (Hypothesis 6, β =  0.14; P =  0.008). Meanwhile, the relationship between additive technologies and process innovation is strong (Hypothesis 4, β =  0.67; P <  0.001), as is the relationship between strategies and operational effectiveness (Hypothesis 3, β =  0.54; P <  0.001), and the relationship between process innovation and strategies (Hypothesis 5, β =  0.53; P <  0.001). The relationship between additive technologies and strategies is weak (Hypothesis 2, β =  0.25; P <  0.001), as well as the relationship between additive technologies and operational effectiveness (Hypothesis 1, β =  0.31; P <  0.001).

**Table 4:** SEM results for the aerospace sector.

			Estimate	S.E.	C.R.	P	H
Operational Effectiveness	<-	Additive technologies	0.291	0.040	7,225	***	H1
Strategies	<-	Additive technologies	0.264	0.051	5,169	***	H2
Operational Effectiveness	<-	Strategies	0.479	0.053	9,079	***	H3
Process Innovation	<-	Additive technologies	0.714	0.058	12,265	***	H4
Strategies	<-	Process Innovation	0.615	0.055	11,138	***	H5
Operational Effectiveness	<-	Process Innovation	0.123	0.046	2,670	0.008	H6

## 5. Discussion

The comparison (Table 5) between the β of the industry in general and the aerospace sector shows the following results:

**Table 5:** Comparison of SEM results.

			Manufacturing sector (except Aerospace)		Aerospace		
			β	P	β	P	
Operational Effectiveness	<-	Additive technologies	0.10	0.005	0.31	***	H1
Strategies	<-	Additive technologies	0.22	***	0.25	***	H2
Operational Effectiveness	<-	Strategies	0.34	***	0.54	***	H3
Process Innovation	<-	Additive technologies	0.76	***	0.67	***	H4
Strategies	<-	Process Innovation	0.68	***	0.53	***	H5
Operational Effectiveness	<-	Process Innovation	0.52	***	0.14	0.008	H6

For the Aerospace sector, only hypothesis 6 was rejected. Process innovation has no significant relationship with operational effectiveness (β =  0.14; P =  0.008). Meanwhile, there is a strong and close relationship between additive technologies and process innovation (β =  0.67; P <  0.001), confirming the proposals by Steenhuis & Pretorius [83] and Baumers & Holweg [93]. These authors suggest that additive technologies represent an innovation in the organization, affecting its processes incrementally in some industries and radically in others. The results, both for the industry in general and the aerospace sector, indicate that organizations understand the importance of additive technologies in driving innovations in their processes and routines and that these changes are necessary.

On the other hand, changes in organizational processes have consequences for organizational strategies because these changes, aimed at improving competitive positioning and addressing environmental changes, generate shifts in the organization’s capabilities and competencies, thus expanding the strategic horizon [67]. The results of this study confirm this view, as process innovation has a significant impact on strategies, which is valid not only for organizations across all sectors but also for the aerospace sector.

Interestingly, additive technologies have little impact on operational effectiveness and organizational strategies in the aerospace sector. For organizations in other manufacturing sectors, the impact of additive technologies on strategy is low, and there is no impact on operational effectiveness. These results contradict the literature in which additive technologies significantly boost organizational productivity and competitive advantage [38,94]. The reasons for this situation can be manifold. Firstly, Colombian organizations have limited knowledge of this new technology and its benefits, resulting in inadequate processes for implementing such innovations and, consequently, a lack of impact on strategies. Secondly, the initial cost of implementing additive technologies may be too high for organizations, thus having a low impact on operational effectiveness. This result also indicates the low importance of this innovative technology. Another possible cause could be that it is considered a significant change in organizational processes, changes that the organization is not willing to face. In this regard, organizational culture and a rigid, inflexible hierarchy hinder organizational innovation [95,96]. Since additive technologies do not significantly change organizational strategies, they cannot generate the expected impact even if this innovative technology is implemented adequately.

For the manufacturing industry in general, process innovation has a moderate impact on operational effectiveness, as do strategies. In this regard, organizations seem to not capitalize on innovations’ benefits. Although strategies impact operational effectiveness, the results suggest that strategies are focused on only a part of the organization or are not adequately communicated within the organization. The literature suggests a properly implemented strategy significantly increases operational effectiveness and organizational results [97,98]. Similarly, process innovation aims to enhance the organization’s competitive position and, thus, improve results [53,99]. However, it seems that Colombian organizations are not achieving these objectives.

It is also interesting to note that process innovation does not impact operational effectiveness in the aerospace industry. Innovation and change require internal and external components, and innovation becomes challenging with only internal resources [100]. The difference may lie in the fact that the aerospace industry depends mainly on a) connections between various organizations in the aerospace sector and b) conditions imposed by different governments [101]. In Colombia, cooperation with foreign and local organizations may be hindered by the high degree of regulation in the sector. Similarly, the necessary controls on suppliers hinder changes within the organization, and organizations thus remain relatively static in their processes that have worked [102]. Therefore, it is unsurprising that aerospace sector organizations have difficulties changing processes and routines, thus improving their operational efficiency. On the other hand, process innovation also impacts aerospace organizations’ strategies, although this impact is not very strong.

Another result is that the impact of strategies on operational effectiveness in the aerospace industry is more significant than in the manufacturing industry as a whole. The aerospace industry demands a high degree of product and process safety. From the five performance objectives of operational effectiveness (flexibility, costs, reliability, quality, and speed), quality and reliability are particularly significant in this industry, as a failure could result in the loss of human lives. Consequently, organizations in the Aerospace sector define strategies to increase operational effectiveness. These strategies may include Big Data analysis, standardized processes and routines, stringent quality controls, and the design of reliable and durable products [103–105]. In this manner, organizations in the sector ensure that the products they offer meet the requirements of their customers and that customers can receive a quality, reliable product that meets their needs. Hence, the standardization of processes and routines is essential, given the increasing complexity and costs of aerospace systems, and process innovations are carefully planned and executed [106]. Due to lower risks and investments, organizations may be more inclined to implement innovations in their processes and routines in other industries.

The differing SEM results for the aerospace and non-aerospace sectors in this study highlight the distinct dynamics and operational realities of these industries. In the aerospace sector, additive technologies show a more significant direct impact on operational effectiveness compared to the manufacturing sector. This stronger relationship is likely due to the aerospace industry’s reliance on advanced materials and precision engineering, which align closely with the capabilities of additive manufacturing. The ability to produce lightweight, intricate, and high-performance components makes additive technologies a critical innovation in Aerospace, where weight savings directly translate to fuel efficiency and cost reductions. Conversely, in the general manufacturing sector, additive technologies do not directly impact operational effectiveness. This discrepancy can be attributed to the sector’s broader range of products and processes, many of which do not require the level of precision and material efficiency that additive manufacturing offers. Additionally, traditional manufacturing methods may already be optimized for mass production, reducing the relative advantage of adopting additive technologies.

Process innovation also plays a contrasting role in these sectors. In the general manufacturing sector, process innovation significantly impacts operational effectiveness, indicating that manufacturers are leveraging new processes to improve efficiency, reduce costs, and enhance product quality. This reflects a readiness to adapt and innovate within existing frameworks, utilizing process improvements as a pathway to competitive advantage. However, in the aerospace sector, process innovation does not significantly impact on operational effectiveness. This outcome may stem from the stringent safety and quality requirements in Aerospace, where process changes must undergo rigorous validation and are often implemented cautiously. The high stakes associated with failure in Aerospace make organizations less likely to adopt radical process innovations, focusing instead on incremental improvements that prioritize reliability over efficiency gains.

The influence of strategies on operational effectiveness also varies between the sectors. Strategies have a stronger impact on operational effectiveness in the aerospace sector, reflecting the industry’s emphasis on structured planning, risk management, and long-term investment in innovation. Aerospace organizations often operate within highly regulated environments and depend on strategic frameworks to ensure compliance, optimize resource allocation, and maintain competitiveness. In contrast, strategies in the manufacturing sector have a moderate impact on operational effectiveness, potentially due to less stringent regulatory requirements and a broader diversity of strategic priorities that dilute their direct influence on operations.

These differences also reflect sector-specific external factors. The aerospace sector’s dependence on global supply chains, government contracts, and collaborations with international organizations creates unique constraints and opportunities for innovation. In contrast, the manufacturing sector often operates with more localized supply chains and faces fewer regulatory hurdles, enabling greater flexibility in adopting process innovations and technologies. Furthermore, the cost of implementing additive technologies is a significant barrier for many manufacturing organizations, particularly small and medium-sized enterprises, whereas the aerospace sector’s higher budgets and critical need for innovation justify these investments.

## 6. Conclusions

This study reveals distinct outcomes for the manufacturing and aerospace industries regarding the relationship between additive technologies, strategies, process innovation, and operational effectiveness. Organizations across sectors should prioritize implementing additive technologies to enhance productivity and gain a competitive edge. These technologies can be integrated through in-house capabilities or outsourcing. However, adopting such innovations necessitates strategic adjustments, as they reshape competencies and operational processes.

For the aerospace sector, innovation models and process routines must be revisited since their current configurations fail to enhance operational effectiveness. A thorough review of process scope, frequency, complexity, and implementation methods is essential to yield positive impacts. Aerospace organizations must also recognize that Industry 4.0 concepts, including additive technologies, induce cross-cutting organizational changes. Adapting to these transformations is critical for survival in competitive markets.

Differences between sectors emerge in their perception and application of innovation. The general manufacturing industry acknowledges the importance of process innovation for operational effectiveness but underutilizes the potential of additive technologies. This indicates an opportunity for manufacturers to capitalize on the benefits of 3D printing, such as reduced waste and improved production flexibility. In contrast, the aerospace sector exhibits resistance to process innovations despite recognizing the value of additive technologies for advanced product development.

Barriers in Colombia, including limited access to technology, skill gaps, and high implementation costs, hinder the full potential of additive manufacturing. Smaller businesses face challenges in acquiring equipment and expertise, while infrastructural deficiencies further exacerbate these issues. Addressing these constraints requires a collaborative effort to improve workforce training, infrastructure, and access to financial support.

The findings emphasize the importance of aligning strategies with operational changes to maximize the benefits of additive technologies. Strategies must foster adaptability and integration of innovations into processes, ensuring that operational effectiveness is consistently enhanced. For the aerospace sector, strategic focus on reliability and quality is crucial due to safety-critical requirements, while manufacturing industries must overcome traditional production biases to embrace additive technologies fully.

### 6.1. Theoretical implications

Adopting additive technologies in the aerospace and manufacturing sector extends to redefining design paradigms, evolving supply chain theories, and advancing quality control and safety theories. These theoretical underpinnings are essential for comprehensively understanding the transformative potential and challenges of incorporating additive technologies.

An essential finding of this study is that strategies are shaped by technological innovations such as additive technology and process innovation. Today, an organization must adapt its strategies to the demanding changing technologies.

### 6.2. Practical implications

South American nations have historically experienced industrial deceleration, a phenomenon primarily attributable to their reliance on affluent economies and the implementation of inadequate economic policies. Countries like Brazil, Argentina, Chile, and Colombia encounter formidable challenges in their quest to enhance their economic conditions. These challenges stem from heightened global competition and a notable absence of the adoption of lean practices that could substantiate significant advancements within their manufacturing sectors.

Given the Colombian infrastructure and topography, additive technologies are an adequate alternative to enhance competitiveness. Several benefits derive from the introduction of additive technologies (3D print). Firstly, the ability to create complex geometries with precision allows for the production of intricate components that were previously unattainable through traditional manufacturing methods. This facilitates product design innovation and customization to meet specific customer requirements. Secondly, additive technologies have the potential to streamline supply chains by enabling localized and on-demand production, reducing the need for extensive inventories and long-distance transportation of goods. That can lead to cost savings and a more sustainable approach to manufacturing. Furthermore, adopting additive technologies can enhance manufacturing efficiency through reduced material wastage and improved resource utilization. As a result, manufacturers can achieve greater cost-effectiveness and environmental sustainability. Moreover, increased speed and flexibility in additive manufacturing processes can accelerate product development cycles, allowing rapid prototyping and iteration. This agility can be a competitive advantage in industries where time-to-market is critical.

For the aerospace sector, additional benefits arise with implementing additive technologies. The aerospace sector is characterized by stringent requirements for lightweight, complex, and high-performance components, and additive manufacturing offers unprecedented advantages in meeting these demands. By utilizing these technologies, aerospace manufacturers can produce intricate, lightweight structures with reduced material waste, leading to significant weight savings in aircraft and spacecraft. This has the potential to enhance fuel efficiency and reduce emissions, which is a paramount concern in environmental sustainability and regulatory constraints, as well as enhance competitive advantage.

Colombian organizations can strategically embrace additive technologies to enhance operational effectiveness by adopting targeted strategies tailored to their unique context. First and foremost, fostering a culture of innovation and providing employee training on additive manufacturing techniques is crucial. Investing in research and development units dedicated to exploring and integrating additive technologies will enable organizations to stay at the forefront of advancements. Collaborations with local educational institutions and industry experts can facilitate knowledge exchange. Additionally, establishing partnerships with specialized additive manufacturing providers or creating in-house expertise can streamline implementation. Developing a phased implementation plan that starts with pilot projects allows organizations to test and refine additive technologies before scaling up. Integrating these strategies ensures that Colombian organizations not only leverage the operational benefits of additive technologies but also navigate potential challenges effectively, ultimately driving sustained growth and competitiveness.

### 6.3. Limitations

We used a convenience sample based on the Colombian Air Force supplier and the organizations that participated in this study. Although the research is limited to Colombia, and therefore, generalization is questionable, it provides a starting point to understand the impact of process innovation and additive technologies in a competitive landscape in other Latin-American countries. Various predictive models may be examined to attain a more comprehensive examination of the behavior exhibited by analogous regional areas when subjected to specific management scenarios.
